# The Vulnerability-Stress-Model—Holding Up the Construct of the Faulty Individual in the Light of Challenges to the Medical Model of Mental Distress

**DOI:** 10.3389/fsoc.2022.833987

**Published:** 2022-05-23

**Authors:** Elena Demke

**Affiliations:** Department of Psychiatry and Psychotherapy, Brandenburg Medical School, Immanuel Klinik Rüdersdorf, Rüdersdorf, Germany

**Keywords:** psychiatrization, vulnerability-stress-model, psychoeducation, movement of (ex-)users and survivors of psychiatry, medical model

## Abstract

In the late 1970s, the course seemed to be set for a reconciliation of the controversy around the somatic vs. the social nature of mental distress. The biopsychosocial model and the vulnerability-stress-model were influential agents in this move, but a medicalized somatic view on mental distress persisted nonetheless. The reasons for this persistence are complex, and naturally include questions of structural power. However, the adherence to a certain fundamental framing of a problem may continue to be transmitted not only out of conviction, but also unwittingly. The vulnerability-stress-model allowed those who used it to effectively stick to the implications of a medicalized somatic view of the faulty individual who falls ill, while also allowing them to believe they integrated the social dimensions of the problem. A close reading and hermeneutical interpretation of the text by Zubin and Spring (1977) and an analysis of its use in psychoeducation serve as a case study in this respect. The vulnerability-stress-model (simply called “vulnerability model” by Zubin and Spring; more often “stress-vulnerability model” by English speaking recipients, and “vulnerability-stress-model” by German authors) seems to have been a success story: since its publication by Zubin and Spring (1977), it has been the point of reference for numerous scholarly and popular (“psychoeducational”) adaptations. It was soon extended from the diagnosis of schizophrenia to various psychiatric diagnoses, understanding mental distress as the result of a trait/state-interaction in the shape of “deviant coping patterns” (Zubin and Spring, p. 112). Recipients appraised the integration of environmental and dispositional factors, some of them opposing the supposed originally integrative intention of the VSM to reduced applications of it (Schmidt, 2012). However, it can be argued that this integration is a matter of rhetorics rather than argumentative essence. Their argument which significantly depends on the use of metaphors, as well as their referencing amounts to a confirmation of a medicalized view on mental distress and a dismissal of the role played by societal factors. Applied to psychoeducation, this paradoxical combination reinforced a view of the persons in question as individually vulnerable, rather than socially wounded. The consequences in terms of what appears as remedy are significant and contribute to turning individual difference into disability.

## Introduction

J. Zubin and B. Spring were both psychologists, the former being a senior researcher specializing in questions of biometrics, pharmaceutical issues and the diagnostics of schizophrenia, and the latter his doctoral student.

The central argument of their vmodel (Zubin and Spring, 1977) runs along the lines of state-and-trait-interaction: People with an enduring disposition (trait), called vulnerability, are more strongly affected by events that elicit stress (state). The higher the vulnerability the lower the level of stress, resulting in episodes characterized as illness, which in close succession may present themselves as a seemingly permanent condition that is then called schizophrenia.

The authors put forward this concept of vulnerability under the title of a “new view on schizophrenia” in a contribution to the “Journal of abnormal psychology.” The very name of the journal, the talk by the authors of “faulty reaction to life's exigencies” (Zubin and Spring, 1977, p. 112), as well as of “deviant coping patterns” (ibid.), strike today's reader as witnesses to an era in which derogatory turns of phrase applied to those judged to divert from psychosocial norms were the accepted scholarly norm [In fact, only recently, the editors announced that the journal will be renamed, citing that “the terms abnormal and abnormality now are pejorative tropes” (MacDonald and Watson, 2021, p. 1)]. Historically seen, it is not the pejorative labeling which appears to be new, but rather the societal rules banning its prominent use.

Zubin and Spring's (1977) work has been one of the foundations for a framing of mental distress which has been most influential since: the proclaimed integration of biological and societal aspects, in this instance understood as the interaction of vulnerability and stress, hence vulnerability-stress-model (VSM) or diathesis-stress-model. The VSM has been considered an “extremely useful model” (e.g. Goh and Agius, 2010) for decades, even by proponents of alternatives to traditional psychiatric care. For instance, Luc Ciompi, one of the founding figures of the soteria—which focuses on interpersonal and authentic (rather than professionally trained) ways of encounter as the road to recovery—considered his own work to be building upon the VSM (http://www.ciompi.com/de/schizophrenie.html, last accessed: 5/02/2022).

The VSM has been a popular point of reference in the various research contexts in which it has been theoretically modified and elaborated (e.g. Nuechterlein and Dawson, 1984; Nuechterlein et al., 1994; Hankin and Abela, 2005; Ferriter, 2019), in the training of medical students (e.g. Broerman, 2017), and in particular, in the medico-paedagogical contexts of German Psychoedukation (psychoeducation) for patients and their relatives (e.g. Bäuml, 2005; Bäuml and Pitschl-Walz, 2007). Thus, this article which appears merely historical on one hand, and of lasting influence on the other, is worth a closer look: Which contemporary debates do the authors take up? What is their specific line of argument, and what sources or evidence do they rely on? And what was the wider socio-political context in which it was published?

The VSM is related to a long-standing debate in both the philosophical and medical traditions: the question of the explanatory power of “nature vs. nurture,” given that vulnerability (or diathesis) is understood as mainly inborn, and stress as the effect of later occurrences. The model thus promises to solve a historical conundrum with respect to what was considered as mental illness, specifically to do with the diagnosis of schizophrenia. Concerning so-called severe mental illness, and despite national differences in intensity and exclusiveness (Bernet, 2013), psychiatry had had a long trajectory of stressing the “nature” aspect from the late 19th century onwards. In opposition to it, there stands a centuries-old common senses—as put succinctly, for example, by the German classicist dramatist G. E. Lessing in the 18th century: “Whoever doesn't lose his mind over certain things has no mind to lose” (in the play Emilia Galotti, IV.7). It has been frequently reformulated since, in particular by psychiatric survivors throughout the 20th century, who insist that suffering is caused by “broken hearts, not broken brains” (Sen, 2017), thus emphasizing the “nurture” (i.e. the environmental) side of the problem. Without naming it in these abstract terms, the VSM takes a stance on this fundamental conflict.

Modeling experiences diagnosed as schizophrenia as an outcome of the interaction of disposition and environmental factors was not new in 1977. Zubin and Spring themselves refer to Meehl (1962) as a predecessor arguing along similar lines, and so did Bleuler (1963). The term “vulnerability” had also been used in the context of describing psychopathology before (Beck, 1967). This puts additional stress on the question: why have Zubin and Spring (1977) become such a popular point of reference, in particular in psychoeducation? What is the socio-historical significance of their formulation of the VMS in the mid-1970s?

## Revisiting the Original Publication of the VSM

### A Close Reading

#### Structure and Rhetoric

In order to tackle these questions, the article by Zubin and Spring (1977) will be considered not just as a set of arguments but as a text, the meaning of which is constituted through specific textual features such as the use of rhetorical means and metaphors as well as its intertextuality. These aspects will be analyzed in the vein of a close reading as suggested by literary scholars (Basseler, 2013). Zubin and Spring (1977) start with an overview of the “descriptive psychopathology” and “etiology” of the diagnosis of schizophrenia in just five pages. They move on from passing remarks on Ancient Ayurvedic teachings (ibid, p. 103) and pre-historic times to Kraepelin and recent research, such as the WHO's 1973 pilot study on schizophrenia (ibid., p. 104), paraphrasing these references in ways that imply that what contemporary medicine diagnoses as schizophrenia is in essence a transcultural and transhistorical phenomenon. At the same time, they do not enter into a scholarly debate on this contentious assumption, nor do they reflect upon the fact that, for example, the WHO study was widely read as a challenge to—rather than a confirmation of—Western psychiatric practices.

Their discussion of etiology rests on an earlier scheme of 6 ways of modeling the origins of the experiences diagnosed as schizophrenia (Zubin, 1972), and concentrates on two of these in order to contrast the “ecological” and “genetic” models; however, other models mentioned (developmental psychological, learning psychological et.al.) would have fallen into the same nature/nurture divide. The concentration on “ecological” vs. “genetic,” together with a narrow understanding of the “ecological,” allows the authors to leave out large strands of etiology that question the medical model—of particular interest being the consequences of poverty, abuse and other social hardships. At the same time, they present the conclusion of these passages as if the chosen models were representative combatants in the argument of nature vs. nurture: “Corresponding to the two types of etiological models—the biological and the field theory—there are two major components of vulnerability, the inborn and the acquired.” (ibid., p. 109).

This brief yet biased overview of psychopathology and etiology sets the stage for the VSM with rhetorical fanfare. Stating that “we are abysmally ignorant of the causes” (ibid., p. 105), and that a “formidable impasse” (ibid, p. 108) had been reached, the authors identify “parochialism” (ibid.) as the culprit and thus present the VSM as both a virtuous and historically necessary solution, avoiding “parochialism” and moving on from an alleged impasse.

#### Debate and Intertextuality: Citations and Omissions

While the journal article is obviously a piece of scholarly work, its way of referencing remarkably diverts from the conventions of the genre. On the matter of the conflict of “nature vs. nurture,” the authors treat friends, bystanders and enemies distinctly differently. They never state this openly or explicitly, but their preference for geneticist views is clear. Thus, when contrasting the two chosen models of “ecological” vs. “geneticist” research, they deal with the former by referring to just one metatheoretical study and talking about unresolved methodological challenges. In contrast, geneticist research is represented by five studies, the arguments and findings of which they judge to be “exciting and striking” (ibid. 106).

When actually addressing questions of environmental factors contributing to “mental illness” in other passages (in the broad sense of including social hardships), Zubin and Spring more often than not omit the names of authors. Thus, they mention the problem of stigma, arguing that the VSM might in fact redress this problem, but they do not mention any piece of research. Dedicating space to Ayurvedic texts but not naming contemporary research—most notably, in the case of stigma, the research by their contemporary (Goffman, 1963/1990)—is a remarkable procedure. In effect, it operates as term-dropping—giving the impression that concepts shaped by a critique of the medical model, such as stigma, are dealt with, without seriously engaging with them in actuality. In this way, the authors give the appearance of working broadly and inclusively, while in fact not doing so—the omission of names seems to be systematic. Zubin and Spring conclude their discussion of psychopathology by asking: “Where does this leave the allegation that schizophrenia is a myth?” (ibid., p. 104)—an obvious allusion to Thomas Szasz' well-known book (Szasz, 1961/1974) that, again, omits naming him. Replacing citation and referencing with allusion is unusual in scholarly texts. By doing so, Zubin and Spring present the undesirable author as one not worth naming. Such a strategy turns into an instance of writing-out-of-history when a text becomes canonical. In this way, provocative knowledge can be removed from the realm of acceptable scholarly discourse.

While not naming him, Zubin and Spring come up with two arguments in reply to the Szasz's criticism, according to which mental illness is an invalid construct (or a “myth”): Based on the consideration that medical diagnoses are indeed constructs, Zubin and Spring claim that as long as there is consensus between experts on the identification of “schizophrenia,” and as long as it is considered beneficial for choosing therapies and interpreting research, the construct should be used. Strangely, they neither use the scholarly term for this consensus (reliability) at this point, nor do they mention the fact that the low reliability of “schizophrenia” had been widely discussed—not least in the context of the WHO study of 1973 which they refer to. Even more irritatingly, they combine the psychometric criterion of reliability with a vague criterion of “being beneficial” (for whom?) as bases to defend the use of the construct of schizophrenia, while validity, of course, remains the foremost criterion (and what Szasz gets at is the validity of the construct of “mental illness”), without which the quality of other criteria becomes irrelevant. To put it bluntly: a myth may produce “reliability”—experts may highly agree in recognizing a certain myth and find this myth useful—but this does not stop it from being a myth. Zubin and Spring, as trained psychologists, naturally would have known about the significance of psychometric criteria, which leaves the reader wondering about their motivation for arguing in this way.

When it comes to psychological research traditions which do not immediately address mental distress but have the potential to contribute to overcoming a narrow medical understanding of it, Zubin and Spring mention famous authors and concepts in ways that strip them of their significance for the topic in question. For instance, they refer to Piaget and his terminological differentiating of assimilation vs. accommodation, which is based on his concept of cognitive schemata in learning (Piaget and Inhelder, 1958). When thinking about the “nature vs. nurture” discussion, this belongs to the realm of “nurture,” and the concept of cognitive schemata could help in understanding why some situations pose much greater and more fundamental challenges to some people than they do to others, rather than thinking about persons possessing greater or smaller capabilities in handling them. However, Zubin and Spring use Piaget's terminology in a biologized way. Thus, they describe coping as the intensity of effort that leads to either assimilation or accommodation, and argue that assimilation consists of changing the environment, whereas accommodation refers to the adaptation of the inner state of the organism to the environment.

This irritating use of terminologies in contradiction to and without discussion of the research contexts they stem from is merged with political statements. Thus, Zubin and Spring use their reading of assimilation vs. accommodation to argue that segregating mental patients in asylums had resulted in a useful reduction of stress through assimilation (ibid., p. 114). This argument can be interpreted as an indirect attack at contemporaneous scholars such as Goffman and his work on the dehumanizing effects of institutionalization, as well as on the movement of psychiatric survivors that shed light on the violent character of mental institutions (Goffman, 1961/1990).

A closer look at the references made by Zubin and Spring in conceptualizing the term “stress” confirms this analysis: They do name authorities from cognitive psychology, but rely on biological concepts. For instance, they mention the name of the cognitive psychologist Lazarus, whose work on the role of appraisal in stress had begun to be published in the 1960s (Lazarus and Alfert, 1964; Lazarus et al., 1965), appearing as a theoretical interface of environmental factors and experiences of stress, thus holding the potential to explain the differing effects of environmental impacts on persons without resorting to concepts of inherent otherness. However, when actually setting forth their understanding of stress, Zubin and Spring rely entirely on the biologist concept of stress proposed by Hans Selye, which in turn has been considered a facilitator for the medicalization of stress (Burrows, 2015). Its underlying stimulus-response-schematism leads to a quantitative modeling of the connection of event and resulting stress in the VSM, which relocate the problem in the deficient individual and her limited capacity for processing stress.

#### Foundation of Arguments: Metaphors and More

Zubin and Spring claim that the VSM is based on a “logical factor analysis” (ibid., p. 109) through which they establish a “second order model” based on finding the “common denominator” of all existing models. However, a factor analysis is used in order to study latent non-observable constructs based on observable phenomena. Treating various existing research models as if they were the observable phenomena from which to draw conclusions about the latent construct of schizophrenia is a surprising mixing up of incompatible levels of observation. Furthermore, formally speaking, it is not a logical conclusion to say that if C does not follow from either A or B alone, it must follow from a combination of A and B. And lastly, even such premises are not given anyway: after all, the discussion of the methodological problems in measuring environmental factors does not justify the conclusion that these factors do not account for distress diagnosed as mental illness.

Zubin and Spring use several metaphors to put forward their understanding of vulnerability: they compare it to the strength of a rope which has to hold a weight and might burst (the weight being the metaphor for stress) (ibid, p. 110); to the heart of a person who has suffered a heart attack and then runs a marathon (ibid., p. 112); to the cracks in the surface of the earth of a volcanic field which make an eruption more likely (ibid, p. 117); and they refer to sickle cell anemia in order to argue that environmental triggers may lead to the outbreak of an illness which is, in essence, genetic (ibid, p. 122). These analogies underline their understanding of stress and vulnerability as similar to natural events or somatic conditions. Coping abilities in turn are understood as effort plus competence, conceived in rather mechanical terms in their comparison to the voltage plus equipment of a machine. Accordingly, their suggestions for interventions are: vulnerability cannot be altered by psychological means, but only through psychopharmacology, while strengthening abilities in coping with stress may prevent the actual “breakdown” (ibid., p. 122) of the vulnerable person. Although additionally making provisions at first for a psychological component in vulnerability—talking of “traumas, specific diseases, perinatal complications …that enhance or inhibit the development of subsequent disorder” (ibid., p. 109)—this aspect does not bear on the further development of their argument. The mechanical metaphors may appear intriguing in particular for the educational usages of the model, but they transmit a rather blunt idea of the faulty individual whose inherent makeup needs pharmacological remedy and whose abilities need improvement. At the same time the rhetorics of Zubin and Spring allow educators to believe they are taking trauma and other psychological causes of vulnerability into account. This makes it even harder for the person seeking support to voice their experience: dimensions of being wounded are not conceptualized in this elaboration of vulnerability and stress interaction, and are thus easily overlooked.

### Socio-Historical Context: The Medical Model Under Challenge

The close reading has shown that Zubin and Spring (1977) have a preference for geneticist research, rely on biologist rather than psychological conceptions of stress and emphasize the necessity of a pharmaceutical response to what they see as a largely unchangeable vulnerability. Far from appreciating the impact of social adversity on human well-being, the model reduces adversity to situations that turn a person's assumed inherent and acquired deficiencies into illness. Against this background, the question arises as to why Zubin and Spring (1977) has been so widely accepted and popularized as an integration of genetic and environmental aspects.

Addressing this question with respect to social actors and power structures, and tracing networks and alliances, is beyond the scope of this article. However, looking at it in terms of discourse, understanding the latter as the “rules of the sayable” (Landwehr, 2002) allows a hypothetical answer, considering what Zubin and Spring (1977) contributed to the Specter of accepted narratives on psychiatric care and mental illness. At the time their article was published, the medical model and psychiatric authority relying on it had been massively questioned for more than a decade. Fundamental criticisms had been put forward by a number of social groups: survivors, media, researchers. Researchers refuting the medical model in psychiatry came from outside as well as from within the profession. Two of the most illustrious names of sociology and philosophy of the 20th century—Goffman (1961/1990, 1963/1990) and Foucault (1969/1961, 2008/2003)—stand for the theorizing of the oppressive social function of psychiatric diagnostics and care, and the academic dissemination and international reception of the works was at their height in the years preceding the publication of the VSM. Around the same time, works of psychiatrists that questioned the theoretical foundation and ethical adequacy of their profession enjoyed high popularity, most famously Szasz (1961/1974) on the lacking validity of the construct of mental illness, and British anti-psychiatrists on social conflict leading to people being diagnosed (Cooper, 1971; Laing, 1971). Media—both journalism and fiction—scandalized dehumanizing aspects of psychiatric care, with the iconic example of this trend, the movie “One flew over a cuckoo's nest,” being released in 1975. Strands of social science translated theoretical critique into experimental research (e.g. Rosenhan, 1973). Last but not least, in the early 1970s, the psychiatric survivor movement emerged, first in North America, England and Scotland. Being part of the new social movements, activists not only fought social injustice and discrimination, but argued that the medical model contradicted their demand for self-determination (Alvelo, 2011; Gallagher, 2017).

It is hard to imagine a more fundamental and massive questioning of a medical profession and institution than that of psychiatry during the 1960s and 1970s. If mental distress and the attribution of diagnoses were to be seen as social processes and psychiatric care had proven of little benefit to those speaking out about their experience receiving it—how could medical authority on psychological distress be upheld? Narratives framing the latter as including social and environmental aspects were needed to invalidate those criticisms. After all, “The view of mental disorders as non-biological psychosocial problems [had become] the source of anti-psychiatric arguments.” (Rzesnitzek, 2013, p. 4).

Zubin and Spring were aware of this contemporary challenge. As has been shown, they avoided naming critics and entering into a discussion, making allusions instead. However, in one instance, they addressed historical circumstances explicitly: “In recent years there has been great concern with the civil rights of patients suffering from mental disorders … there is growing suspicion that the consequences of being labeled and stigmatized as mentally ill may be far reaching, dehumanizing, and injurious to civil rights. In the final analysis, attacks have often focused on the so-called medical model…” (Zubin and Spring, 1977, p. 121). They argued pragmatically, downplaying criticisms and suggesting that the construct of vulnerability might serve as a more acceptable label: “The vulnerability label is perhaps easier to accept and live with, since it presages a timelimited episode from which the patient will … recover” (ibid., p. 121).

Given the rhetorical focus of the article, and the mix of biologist preferences and integrative claims by the authors, it appears plausible that the VSM was successful as just this: a label easier to accept—and to apply—offering practitioners, users and even the wider society a narrative of a psychiatry which had taken into account the role of social adversity in understanding and dealing with mental distress, while not changing much in essence. After all, the medicalized view on mental distress persisted (Read et al., 2009).

### Application and Consequences: Psychoeducation

Medico-pedagogical publications, proliferating in Germany under the name of “Psychoedukation,” follow rather traditional didactic underpinnings, focusing on the dissemination of preconceived knowledge, rather than embracing an understanding of competence which emphasizes multiperspectivity and the transparency of controversial and constructionist dimensions of knowledge (Reusser, 2014). In “Psychoedukation,” expertise is allocated one-sidedly to the medical professional, and a broader view on scholarly and societal approaches to mental distress is not integrated. Not making the addressee aware of the fact that academic knowledge is often controversial is a deliberate choice: “The publication of professional pieces of advice and opinions that are partly contradicting each other is very confusing and irritating for the service user” (Bäuml and Pitschl-Walz, 2007, p. 41). The realm of decision making for service users thus is limited to discussing matters of modifying the doses of medication (Bäuml and Pitschl-Walz, 2007).

In a dissertation dedicated to the critical analysis of psychoeducational approaches in Germany, Schmidt (2012, p. 37) identified the development of a “functional concept of the disease” as one of major goals of “Psychoedukation.” Such an aim precludes the option that the addressee reaches a non-medical definition of her distress as a possibly functional concept based on her mature decision. Declaring “Psychoedukation” as aiming to involve “patients” as “mature partners” (Bäuml and Pitschl-Walz, 2007) in decision making and treatment is a modification in wording that can be observed—but method and content prove the contrary (see Bäuml and Pitschl-Walz, 2007, cf. Bäuml and Pitschel-Walz (2005)). Thus, the expertise of the person to be “psychoeducated” is reduced to applying the authoritative model to one's specificities, i.e. to identify stressors and coping mechanisms, rather than to judge the usefulness of the model for one's own life, taking into account its social and political implications.

These problematic and disempowering aspects of the VSM have been attributed to the fact that it has been stripped of “its original integrative character,” leading to a one-sided focus on “somatic explanation,” as well as “a superficial conception of stress” (Schmidt and Körtner, 2014, p. 241). However, as the close reading of Zubin and Spring (1977) has shown, the reductionism of a simplified understanding of stress and a focus on somatic etiology are not so much a distortion, but genuine characteristics of the original publication.

In psychoeducation these characteristics are transformed into immediate advice. While trusting medical expertise and taking medication as prescribed may appear as the usual goal of a desirable doctor-patient-relationship, there is something different at stake in the context of mental distress. The concept of a given “vulnerability,” deduced from a questionable “logical factor analysis,” concerns the framing of a one's own personality and biography. Should one, for example, struggle to address experiences of abuse, poverty or discrimination, get angry at the social injustices leading to such adversities, and perhaps even engage in activism to fight them? Or should stress be avoided, since it may lead to “illness”?

This is not a rhetorical question or one of politically instrumentalizing mental distress. Rather, it arises from observing a repeated feature in testimonials by psychiatric survivors: Embracing the stress that comes along with addressing trauma and adversity becomes part of the personal road to well-being, and these roads have to be discovered or even fought for against professional advice, as long as said professionals follow the implications of the VSM (e.g. Boevink, 2017; Brosnan, 2017).

A simplified understanding of stress is disseminated through illustrations, diagrams and textual explanation. Illustrations resort to metaphors, often that of buckets that are meant to symbolize the capacity of the person to tolerate stress and which overflow when more water enters than can be contained (e.g. Mediclin, 2018; Wirtz, 2021; Woodward, 2021; Patientenbroschüre, n.d.). The use of cross-section drawings underlines not only the technical character of the illustrations but acts as a way of looking inside, of seeing the otherwise invisible—the differing volume of buckets which look the same from outside. The need for an expert's gaze to recognize the internal condition of the bucket—as well as the notion that insufficiency may come unexpectedly—are additional elements of this iconography. It is also framed by its closeness to depictions of brains: photos alluding to neuroimaging (Mediclin, 2018) or drawings of a cortex (Patientenbroschüre, n.d.).

The message is clear: some people can take in less stress than others and the explanation for this difference lies with those experts who can look into structures invisible to the layperson. This impairment is to be tackled by taking neuroleptics, as set out already by Zubin and Spring (1977) and reinforced by subsequent psychoeducation.

However, the focus on pharmaceutical compliance is not only a consequence of the VSM and its modeling of vulnerability as a defect that can best be addressed by medication, but it is also an expression of the direct influence of pharmaceutical companies that sponsor publications (e.g. Bäuml and Pitschl-Walz, 2007, p. 4) or training for those offering psychoeducation (e.g. http://spi-paderborn.de/2018/03/psychoedukationsworkshop-des-spi-mit-prof-dr-baeuml/, accessed: 10/11/2021). However, the influence of pharmaceutical companies has been made more visible in recent years at least. As an example, a psychoeducational website sponsored by a pharmaceutical company, and produced in collaboration with the renowned Hamburg university hospital, changed its URL from www.psychose-wissen.de (last accessed: 5/6/2018; the title of the URL translates “knowledge on psychosis”) to www.janssenwithme.de (last accessed: 10/11/2021). But boundaries between “Psychoedukation” and pharmaceutical advertisement remain blurred: the pharmaceutical company Janssen runs the website “Schizophrenie 24 x 7,” and advertises it as a “useful offer for first information on this mental illness” [https://www.presseportal.de/pm/16998/3834936]—in fact offering mainly “education” on the inevitable necessity of taking neuroleptics.

Having concluded from the analysis so far that the adoption of mechanical metaphors that is characteristic of the VSM in psychoeducation contributed to the furthering of a medicalized understanding of mental distress in medico-pedagogical publications in Germany, it is worth taking a look at examples from recent psychoeducation in the UK, which bears witness to a stronger research tradition on the role of social adversity, and a more inclusive approach to (ex-)user knowledge (e.g. Longden and Read, 2016). Here, psychoeducational publications can be found which fundamentally divert from a narrow focus on compliance with psychopharmaceutical intervention and encourage users of psychiatry to find individual ways of coping, including an appreciative approach to voice-hearing (e.g. Woodward, 2021). However, the metaphor of the “stress bucket” persists. In these contexts, it is used to illustrate the need to monitor one's intake of stress and to think about ways of “releasing” it, while being formulated with a clear focus on social adversity such as exposure to bullying and bereavement. Examples from online self-help show the metaphor being removed from contexts of marking individual differences altogether by serving as an illustration to reflect on the components of any human experience of stress—leaving out allusions to “buckets” of different qualities in containment, and hinting at the fact that the experience of stress (rather than the containing qualities of “buckets”) is individually unique (Liggins, 2021).

However, with respect to extreme mental distress and the suffering it involves, it might be useful to avoid mechanical metaphors altogether. After all, mechanics can hardly help in conceptualizing the paradoxical, which is characteristic of human experience—including the experience of suffering. Thus, to persons who have experienced extreme adversity such as abuse on a regular basis, exposure to a peaceful and caring environment may result in massive stress. Potential helpers—even those willing to consider social factors causing distress—run the risk of failing people, of being unable to understand the kind of support that is needed, and of staying unaware of the nature of the challenges when holding mechanical metaphors of stress in their minds, and looking for the quantifiable universal stressors psychoeducation tends to model.

## Discussion

The model proposed by Zubin and Spring (1977) represents less an integrative approach and more a defense of the medical model and its reliance on the construct of the deficient individual. This finding is in contrast to the rhetorical claims made by the authors and to the reputation the text enjoys. In psycho-education, in particular in its German version of “Psychoedukation,” this mix of integrative rhetorics and biologist essence supports narratives which are dis-abling for the individual seeking support: The assumption of inherent vulnerability diverts the attention from the gravity of actual wounds, which would have to be taken seriously in order to open up empowering avenues such as fighting for one's rights and against discrimination, victimization and other grievances that are known to make people unwell. Mechanical images about a person's ability to take in “less” stress additionally promote this disempowering and finally disabling approach.

Seen socio-historically, it appears plausible that—published in the late 1970s—a narrative that separated the concern with (psycho-)social grievances from the realm of fundamental criticisms of psychiatric pathologization would have been successful. After all, it suggested that social grievances had been integrated into psychiatric theorizing and practice while allowing for a continued reliance on core elements of the medical model such as the focus on the inherently deficient individual and mandatory pharmaceutical intervention. However, in order to fully understand the genealogy of this persistence and the role of the VSM in it, further research is needed: It would be worthwhile to compare the reception of texts such as Engel (1977) and Zubin and Spring (1977) and also reconstruct the role of networks and power structures in the emerging popularity of the so-called “bio-psycho-social” approach while relating it to a critical appraisal of the seriousness dedicated to “psycho-social” aspects in its application. After all, the question needs to be answered: Why a period of such fundamental critique of psychiatric theory and practice as the one seen in the 1960s and 1970s failed to prevent further psychiatrization of Western societies.

## Author Contributions

The author confirms being the sole contributor of this work and has approved it for publication.

## Funding

This work was supported by Brandenburg Medical School Theodor Fontane. We acknowledge funding by the MHB Open Access Publication Fund supported by the German Research Association (DFG).

## Conflict of Interest

The author declares that the research was conducted in the absence of any commercial or financial relationships that could be construed as a potential conflict of interest.

## Publisher's Note

All claims expressed in this article are solely those of the authors and do not necessarily represent those of their affiliated organizations, or those of the publisher, the editors and the reviewers. Any product that may be evaluated in this article, or claim that may be made by its manufacturer, is not guaranteed or endorsed by the publisher.
